# Expression of neddylation-related proteins in melanoma cell lines and the effect of neddylation on melanoma proliferation

**DOI:** 10.3892/ol.2014.1953

**Published:** 2014-03-07

**Authors:** FANG CHENG, RUNZHI HE, LEI ZHANG, HUI LI, WEI ZHANG, XIAOLIN JI, FANTING KONG, JIANFANG SUN, SHUBO CHEN

**Affiliations:** 1 Department of Dermatology, Affiliated Xingtai People’s Hospital of Hebei Medical University, Xingtai, Hebei 054001, P.R. China; 2 Department of Neurosurgery, Affiliated Xingtai People’s Hospital of Hebei Medical University, Xingtai, Hebei 054001, P.R. China; 3 Dermatology Hospital of Jiangxi Province, Nanchang, Jiangxi 330000, P.R. China; 4 Institute of Dermatology, Chinese Academy of Medical Sciences and Peking Union Medical College, Nanjing, Jiangsu 210042, P.R. China; 5 Department of Surgical Urology, Affiliated Xingtai People’s Hospital of Hebei Medical University, Xingtai, Hebei 054001, P.R. China

**Keywords:** ubiquitination, neddylation, melanoma, apoptosis, cell cycle

## Abstract

Neddylation promotes the process of ubiquitination, which plays a critical role in the degradation of numerous proteins, including cell cycle and apoptosis regulators. In our previous study, an increase in neddylation was identified in melanoma cell lines. In the present study, the upregulation of neddylation was detected in melanoma tissues which confirmed the results of our previous study on melanoma cell lines. To explore the mechanism by which the process of neddylation was increased, the enzymes that regulate the process were investigated. These neddylation-related regulatory enzymes are potential targets for melanoma therapy. Downregulation of UBA3, a subunit of the E1 enzyme, by RNA interference caused cell cycle arrest at G0^/^G1 in the M14 cell line. In addition, cyclin D expression declined, whereas p27, p21 and bax expression increased. These findings suggest that interfering with the neddylation pathway may decrease the proliferation of melanoma through the modulation of cell cycle regulators and apoptosis promoters.

## Introduction

Ubiquitination and neddylation are necessary for a number of biological processes and have been implicated in numerous diseases, particularly in cancer (1,2). The process of ubiquitination has been identified as the mechanism that labels proteins for degradation by the 26s proteasome (3). Proteins degraded by the ubiquitin-proteasome pathway (UPS) are known to be involved in numerous biological processes, including apoptosis (4,5), cell cycle regulation (6) and receptor regulation (7). Conjugation of ubiquitin to its substrate requires three enzymes, ubiquitin-activating enzyme E1, ubiquitin-conjugating enzyme E2 and ubiquitin ligase enzyme E3.

The neural precursor cell-expressed developmentally downregulated 8 (NEDD8) protein is 60% identical to ubiquitin and also conjugates to target proteins (8). This process is termed neddylation and is similar to ubiquitination. NEDD8 genes are translated into non-conjugatable precursors, which contain additional C-terminal residues. Ubiquitin carboxyl-terminal hydrolase isozyme L3 (UCHL3) then cleaves the C-terminal of NEDD8, giving rise to mature NEDD8. Subsequently, NEDD8 is bound to target proteins by a series of enzymes. NEDD8 is activated by the E1 enzyme (APPBP1/UBA3 heterodimer), whereby a thioester bond is formed with the cysteine residue of the UBA3 subunit. Following this, NEDD8 is transferred to the E2 enzyme (UBC12) and, with the involvement of the E3 enzyme, NEDD8 remains bound to E2 and binds to the substrates via its carboxy-terminal glycine (9). Using UCHL3, NEDD8 may be hydrolyzed from the substrates.

Members of the cullin family, which serve as the scaffold proteins of the Skp/Cul/F-box (SCF) complex, are the main substrates of NEDD8 (10). The SCF complex is the core subunit of E3 for ubiquitination; therefore, neddylation may regulate the degradation of proteins by modulating the process of ubiquitination. Ubiquitin, which labels proteins involved in apoptosis and cell cycle regulation, has been implicated in many cancer types (11,12). The anticancer activity of bortezomib, which targets the UPS, has been approved by the FDA (13,14). However, proteasome inhibition, which irreversibly arrests the UPS pathway, causes protein turnover to become disordered (15,16). Study is now beginning to focus on alternative targets that specifically inhibit the growth of tumors through UPS modulation (16). Therefore the neddylation pathway has attracted attention as modulation of such has a higher safety profile which avoids removing the UPS entirely and prevents the conversion of protein from being entirely suppressed.

Neddylation has been studied in certain cancer cells; results have revealed that in highly proliferative cell lines the levels of conjugated NEDD8 expression were high (17). In our previous study, it was observed that the upregulation of neddylation was closely associated with the proliferation capacity of melanoma cell lines (18). In the present study, expression levels of conjugated NEDD8 were examined in melanoma tissues to confirm the results of our previous research. To investigate the mechanisms by which upregulation of neddylation affects proliferation of melanoma cell lines, the expression levels of enzymes that regulate the process were identified and the influence of neddylation on the cell cycle of M14 cells was investigated. The levels of cell cycle regulators and apoptosis-related proteins were also analyzed. To inhibit the neddylation pathway, without altering the function of free NEDD8, UBA3 was chosen as the target for interference (17,18).

## Materials and methods

### Clinical samples

Seven patient samples of melanoma tissue were obtained from the Chinese Academy of Medical Sciences and Peking Union Medical College (Nanjing, China). All cases of melanoma were diagnosed pathologically, and patients had not been treated with chemotherapy or radiation. In the seven samples of melanoma, the majority were of acral location; however, one sample originated from the thigh. Clark classification levels are shown in Table I. All melanoma tissues were stored at −70°C. Patients provided informed consent and this study was approved by the Medical Ethics Committee of the Institute of Dermatology, Chinese Academy of Medical Sciences and Peking Union Medical College.

### Cell lines and culture conditions

The human melanoma cell line, A375, was obtained from Xijing Hospital (Shanxi, China). M14 and MV3 human melanoma cell lines were purchased from KeyGen Biotech Co. Ltd. (Nanjing, China). The three cell lines, A375, M14 and MV3, were cultured in Dulbecco’s modified Eagle’s medium (DMEM) with 10% fetal bovine serum (both purchased from Gibco-BRL, Eggenstein, Germany) at 37°C with 5% CO_2_. The normal melanocytes were separated from the prepuce, obtained from healthy adult males who had undergone circumcision and had been admitted to the Institute of Dermatology, Chinese Academy of Medical Sciences & Peking Union Medical College, and cultured with M254 medium (Gibco-BRL) containing human melanocyte growth supplement (Gibco-BRL) at 37°C with 5% CO_2_.

### Western blot assay

Following two washes with phosphate buffered-saline (PBS), total proteins were extracted from cells and tissue masses using lysis buffer (50 mM Tris-HCl, pH 8.0; 150 mM NaCl; 0.02% NaN_3_; 0.1% SDS; 1% Nonidet P-40; 0.5% sodium deoxycholate; 100 mg/ml phenylmethylsulfonyl fluoride; 1 mg/ml aprotinin; 1 mg/ml leupeptin and 1 mg/ml pepstatin A; KeyGen Biotech Co. Ltd.). Protein concentration was determined by a spectrophotometer (UV-3540;. Seventy micrograms of protein, separated by 10% SDS-polyacrylamide gel electrophoresis (Bio-Rad, Hercules, CA, USA), was transferred onto a polyvinylidene flouride (PVDF) membrane (Pall Corp., New York, NY, USA) by electroblotting. Subsequently, the PVDF membrane was stained with polyclonal anti-goat NEDD8 antibody and polyclonal anti-rabbit bax, p21, p27 and cyclin D antibodies (Santa Cruz Biotechnology, Inc., Santa Cruz, CA, USA), and β-actin antibody (Santa Cruz Biotechnology, Inc.) was used as the loading control. Peroxidase-conjugated anti-rabbit IgG (Santa Cruz Biotechnology, Inc.) was used as a secondary antibody and visualized using a enhanced chemiluminescence kit (Santa Cruz Biotechnology, Inc.). Glyco Band-Scan software 4.5 (Prozyme, San Leandro, CA, USA) was used to quantify the relative quantity of protein.

### Semi-quantitative real-time polymerase chain reaction (PCR)

Total RNA was extracted from the A375, M14 and MV3 cell lines and the normal melanocyte cell lines, according to the manufacturer’s protocol. Total RNA was reverse transcribed into cDNA using the Reverse Transcription system (Promega, Madison, WI, USA). The purity of RNA was determined by measuring the ratio of absorbance at 260 and 280 nm, regulating the optical density between 1.8 and 2.1 ensured a high purity. The sequences of specific primers for each gene, synthesized by GenScript (GenScript USA Inc., Piscataway, NJ, USA), were as follows: Forward, 5′-ACAGTGGCAAGCAGATGAATGA-3′ and reverse, 5′-ATGAGCGACAGGGTAAAGAGGT-3′ for NEDD8; forward, 5′-GCGAGGAGCCGGAGAAGAAAAG-3′ and reverse, 5′-TCGAAATCAGGGTGTGTGAAGG-3′ for UBA3; forward, 5′-GTTTTAAGGTGGGCCAGGGTTA-3′ and reverse, 5′-GGTTGGGCTCCAAGAAGAGATA-3′ for UBC12; forward, 5′-CTACATCCTAACTGGCAATTCGTT-3′ and reverse, 5′-GCGATTTTATTTTTTCTTCCTCT TCT-3′ for UCHL3; forward, 5′-CATTTTGGATTTTAGCTCG TGC-3′ and reverse, 5′-ATCTTTCTTTGCTTTTTCACGG-3′ for APPBP1; forward, 5′-GCAGAAGGAGATCACAGCCCT-3′ and reverse, 5′-GCTGATCCACATCTGCTGGAA-3′ for β-actin. The reaction system contained: 10 μl 2X RT-PCR quick master mix (Toyobo Co. Ltd., Osaka, Japan), 10 pmol upstream and downstream primer and 1 μl DNA template, made up to 20 μl with purified water. The reaction conditions were as follows: One cycle for 5 min at 95°C; and 40 cycles of 95°C for 15 sec, 60°C for 30 sec and 72°C for 30 sec. Size and quantity of amplified products were determined by 2% agarose gel electrophoresis (BioSun Sci & Tech Co., Ltd., Shanghai, China). The ΔCt value was calculated and fluorescence was analyzed using the thermal cycler’s software package (DA 7600, Da An Gene Co. Ltd., Guangzhou, China). The 2-ΔΔCt value was presented as the relative expression of each gene.

### siRNA transfection

In total, three shRNA fragments against UBA3 were designed and synthesised by GeneScript Corp. (Piscataway, NJ, USA): shRNA-UBA3-1, 5′-GGATCCCGTTCCTCGAGCGATCTGGATTCAAGAGA TCCAGATCGCTAGGAACTTTTTTCCAACTCGAG-3′; shRNA-UBA-3-2, 5′-GGATCCCGAACGAACAAGGCCCA AATCTTCAAGAGAGATTTGGCCTTGTTCGTTCTTTTT TCCAACTCGAG-3′; and shRNA-UBA3-3, 5′-GGATCCCGT GCACGCTGGAACTTTATCTTCAAGAGAGATAAAGTT CCAGCGTGCATTTTTTCCAACTCGAG-3′. Fragments were subcloned into the pRNAT-U6.2/Lenti siRNA expression vector (Invitrogen Life Technologies, Carlsbad, CA, USA). Transfection was performed using FuGene^®^ HD (Roche, Basel, Switzerland) and Opti-DMEM (Gibco-BRL) without antibiotics. To investigate the transfection conditions, cells were seeded in 96-well plates. The final reaction conditions were as follows: 2 μg DNA plus 30 μl FuGene HD in a six-well plate with a volume of 2 ml culture medium and 80–90% cell density. Following two weeks of selection using 1000 μg/ml G418 (Invitrogen Life Technologies), G418-resistant clones were selected and maintained with 500 μg/ml G418 for further analysis. The optimum shRNA sequence for UBA3, analyzed by western blotting, was determined as follows: GGATCCCGTGCACGCTGGAACTTTATCTT CAAGAGAGATAAAGTTCCAGCGTGCATTTTT TCCAACTCGAG (71 bp).

### Cell cycle analysis

The Cell Cycle Detection kit (KeyGen Biotech Co. Ltd.) was used to evaluate the cell cycle of non-transfected M14, M14/shRNAT-U6.2 and M14/shRNA-UBA3 cell lines. Cells (1.0×10^6^) were collected, washed twice with PBS and fixed with 70% ethanol at 4°C for 24 h. After discarding the fixation fluid, cells were washed again and incubated with 100 μl RnaseA for 30 min at 37°C followed by staining with 400 μl PI at 4°C for 30 min in the absence of light. The DNA content was determined by flow cytometry (BD FACSCanto II, BD Biosciences, Franklin Lakes, NJ, USA). Cell cycle distribution was evaluated using ModFit software (Verity Software House, Inc., Topsham, USA). Each experiment was repeated four times independently.

### Statistical analysis

Data are expressed as the mean ± SD. SPSS 13.0 statistical software package (SPSS Inc., Chicago, IL, USA) was used to perform the independent samples t-test on the semi-quantitative real-time PCR analysis and cell cycle analysis data, while the paired samples t-test was performed on the western blot analysis data regarding melanoma tissues and surrounding normal tissues. P<0.05 was considered to indicate a statistically significant difference.

## Results

### NEDD8 conjugation in tissues

In our previous study, it was identified that NEDD8 conjugation was upregulated in melanoma cell lines (18). In the current study, conjugated NEDD8 expression was also demonstrated to be upregulated in melanoma tissue. In a previous study using electrophoresis, it was observed that NEDD8 conjugation may be identified by a series of bands including a dominant band of 90 kDa and a minor band of 66 kDa; such findings are consistent with the molecular mass of NEDD8-cullins conjugates (17). As a result of this, in the current study, 90- and 66-kDa bands were observed.

**Figure 1:**
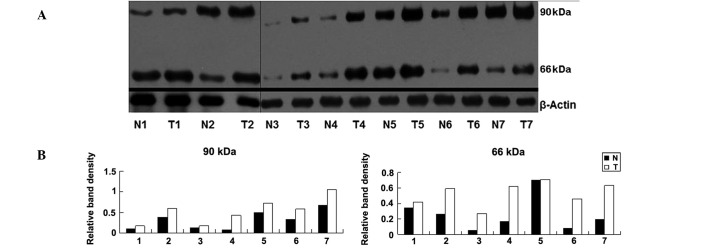
(A) Western blot assay of the NEDD8 conjugation levels in seven paired samples of melanoma (T) and surrounding normal (N) tissues. (B) Column diagram of relative density of NEDD8 conjugation in these tissues. Paired sample t-tests were performed and statistically significant differences were identified between the two groups (t=5.732, P=0.001 at 90 kDa; t=4.132, P=0.006 at 66 kDa). The relative density of NEDD8 to β-actin was as follows: (0.10, 0.17), (0.38, 0.59), (0.03, 0.18), (0.08, 0.43), (0.5, 0.72), (0.32, 0.58), (0.68, 1.06) at 90 kDa; (0.34, 0.42), (0.26, 0.59), (0.05, 0.27), (0.17, 0.62), (0.70, 0.71), (0.08, 0.46), (0.19, 0.63) at 66 kDa.

In this study, NEDD8 conjugation was detected in all seven cases of melanoma and in the normal tissues around them. As shown in Fig. 1, the densities of 90- and 66-kDa bands in melanoma tissues were higher than these in the corresponding normal tissues surrounding them. In Fig. 1, distinct bands of 66-kDa were detected; however in the cell lines analyzed in our previous study (A375, M14 and MV3), this band was absent (18).

### Expression of NEDD8-related proteins in melanoma cell lines and melanocytes

**Figure 2:**
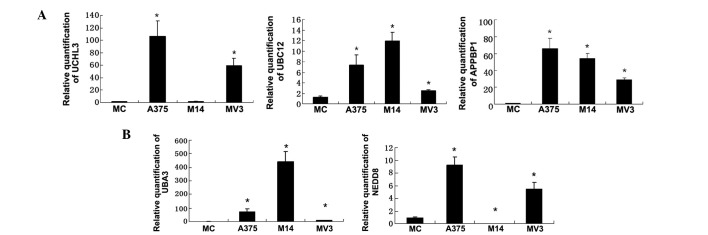
Relative quantification of NEDD8-related proteins in melanoma cell lines and in melanocytes (MC) with β-actin used as the loading control. The relative quantification of (A) UCHL3, UBC12 and APPBP1 and (B) NEDD8 and UBA3, which were reported in our previous study (18). Statistical differences were analyzed with the t-test. Each experiment was repeated three times. (Error bars = standard deviation; ^*^P<0.05, relative to the control of melanocytes).

Gene expression of UBC12, APPBP1 and UCHL3 in three melanoma cell lines and in melanocytes were analyzed by real-time PCR (Fig. 2A). To observe the associations between the NEDD8-related proteins, gene expression data for NEDD8-related proteins, NEDD8 and UBA3, as reported in our previous paper (Fig. 2B) (18), was combined for analysis. It was found that the expression levels of UBC12 and APPBP1 in melanoma cells were upregulated when compared with those in melanocytes. In M14 cells, NEDD8 and UCHL3 expression were both downregulated, but the UBA3 and UBC12 expression was highest compared with those in the A375 and MV3 cell lines and in normal menalocytes.

### Cell cycle arrest of M14 cells following neddylation inhibition in vitro

It has been hypothesized that interfering with the expression of UBA3 may inhibit NEDD8 conjugation and, subsequently, prevent the proliferation of melanoma. However, the mechanisms of this process are unknown. It has been reported that the degradation of cell cycle regulators, such as cyclin D and p21, could be regulated by the UPS (6). In the present study, the effect of NEDD8 conjugation on the cell cycle of M14 cells was investigated.

Percentage distribution of the cell cycle phases in non-transfected M14, M14/shRNAT-U6.2 and M14/shRNA-UBA3 cell lines are shown in Table II. After transfection with shRNA-UBA3, the G0/G1 and apoptotic phases accumulated, whereas the G2/M+S phase declined, when compared with non-transfected M14 and M14/shRNAT-U6.2 cells. The statistical differences between groups were identified to be significant (P<0.05). The increase in G0/G1 phase percentage distribution indicates the inhibition of cell division. These results suggest that inhibition of neddylation in M14 cells may cause cell cycle arrest at G0/G1 phase, promoting apoptosis.

### Effects of neddylation on proteins involved in cell cycle or apoptosis

It was observed that the G0/G1 phase and the apoptotic phase of the cell cycle increased in M14/shRNA-UBA3 cells compared with M14/shRNA-U6.2 and non-transfected M14 cells. To explore the possible molecular mechanisms by which neddylation affects melanoma proliferation, the levels of proteins involved in the cell cycle and apoptosis, including bax, p21, p27 and cyclin D were analyzed.

**Figure 3:**
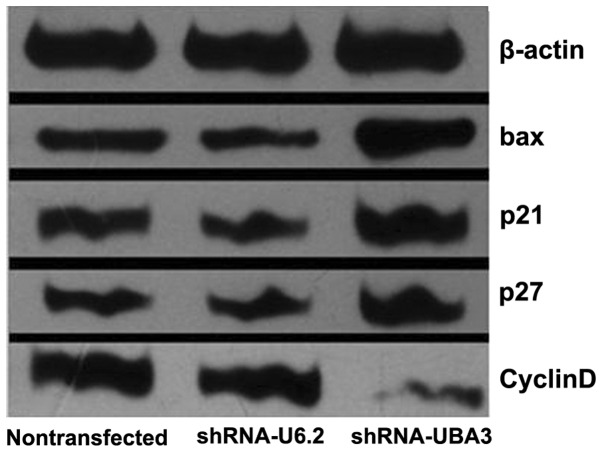
Effect of neddylation on proteins involved in the cell cycle or apoptosis of M14 cells. The relative density of proteins were identified using β-actin as the loading control.

Western blot analysis demonstrated that the levels of bax, p21 and p27 were all upregulated, whereas those of cyclin D were downregulated in M14/shRNA-UBA3 cells, when compared with such levels in non-transfected M14 and M14/shRNAT-U6.2 cells (Fig. 3 and Table III).

## Discussion

Ubiquitination, a post-translational protein modification process, mediates proteasome-dependent degradation of numerous intracellular proteins and has been implicated in a number of cancer types (1). However, complete inhibition of the UPS pathway may cause protein turnover to become disordered (15,19). Recently, there has been increased focus on the neddylation pathway, which is more specialized and may avoid the need to remove the UPS entirely and prevents protein conversion from being suppressed entirely.

Neddylation plays an essential role in cellular survival and is considered to be involved in cancer progression (2,17). In our previous study, the association between neddylation and the proliferation of melanoma was investigated. The results indicated that NEDD8 conjugation was upregulated in melanoma cell lines, and was closely associated with the proliferation of the melanoma cell line, M14 (18).

In the current study, it was identified that there was increased expression of NEDD8 conjugates in melanoma tissues, which indicates the close association between the neddylation pathway and melanoma. The results from seven samples of melanoma showed that NEDD8 conjugation in melanoma tissues was higher than that in the normal tissues surrounding the melanoma. However, individual variations were evident. Certain band densities in normal tissues were higher than the band densities exhibited in the tumor tissues of other groups. For example, in Fig. 1, the band density at 90 kDa exhibited by N7 is higher than that of T3. Biochemical changes may have occurred in the tissues, which appeared normal when observed microscopically; however, they may have contributed to the individual variations observed.

In tissues, not only bands of 90 kDa but also 66 kDa were detected, which is consistent with the study of Chairatvit and Ngamkitidechakul (17), but differs from the results of melanoma cell lines (18). In the current study, the band of 9 kDa, which represents free NEDD8, was negative in tissues, A375, M14 and MV3 cell lines and in normal menalocytes, which was similar to the results of a study by Chairatvit and Ngamkitidechakul (17). These results revealed that the upregulation of NEDD8 conjugation was not accompanied by an increase in free NEDD8 concentration.

The neddylation process may be regulated by a number of enzymes. Expression levels of NEDD8, UBA3 (18), APPBP1, UBC12 and UCHL3 proteins were studied in three melanoma cell lines to determine their association with the upregulation of NEDD8 conjugation. With the exception of NEDD8 and UCHL3 in the M14 cell line, all the other enzymes, which correlated closely with neddylation, were upregulated in the three melanoma cell lines. These upregulated enzymes may be implicated in the increased levels of NEDD8 conjugation observed. As mentioned above, in M14 cells which exhibited the highest levels of NEDD8 conjugation, the expression levels of NEDD8 and UCHL3 were decreased (18). The expression of UCHL3, essential for the NEDD8 precursor to mature, was the lowest in M14 cells, which may be one reason for the lowest levels of NEDD8 expression in M14 cells compared with those the A375 and MV3 cells (21,22). Otherwise, UCHL3 not only makes NEDD8 mature, but also hydrolyzes the NEDD8 conjugation in order to gree NEDD8 (20,21). The UCHL3 decline in M14 may reduce the hydrolysis of NEDD8 conjugation. The relationships among these enzymes are complicated and further studies are required.

In our previous study, proliferation of M14 cells declined following neddylation pathway interference (18). In this study, to investigate the mechanism by which neddylation may affect melanoma growth, cell cycle progression was identified using flow cytometry. The number of the cells in G0/G1 phase and in the apoptotic phase were observed to increase following neddylation interference. Cells never get into the S phase, which is safer than out of control cells coming into the S phase which may lead to the development of cancer. Disturbance the neddylation pathway could arrest cells at the G0/G1 phase but they would be unable to get into the S phase, so that inhibition of mitosis and induction of apoptosis of M14 were onset.

Inhibition of neddylation, which is coupled to the modulation of proteins involved in cell cycle and apoptosis regulation, is thought to induce cell cycle arrest at the G0/G1 phase, consequently promoting apoptosis. Cyclin D, an important growth factor, is essential for cellular fission and cell progression into S phase. Cyclin D upregulation in melanoma is closely associated with melanoma proliferation (22). Inhibition of neddylation, coupled with the decline in cyclin D levels causes cell cycle arrest at G1 phase, leading to apoptosis. Cell cycle progression is also regulated by other CDK inhibitors, including p27 and p21. In the present study, p27 and p21 were identified to be upregulated following inhibition of the neddylation pathway. Overexpression of p27 has been observed to inhibit the formation of the CDK activation complex, preventing cells from entering S phase (23). p21 may promote apoptosis through interaction with DNA repair machinery (24). In this study, the expression of the apoptosis promoter, bax, was also upregulated. Bax is released from mitochondria and cytochrome *c* is released into the cytosol for intrinsic cellular apoptosis (25). In our previous study, it was observed that p53 expression increased following inhibition of neddylation (18). Therefore, G0/G1 cell cycle arrest and apoptosis induction in M14 cells transfected with shRNA-UBA3 may be mediated by the activation of these cell cycle regulators and apoptosis-related proteins.

It has been demonstrated that p53 (26), p21, cyclin D (6) and bax (27) are degraded by the UPS. The upregulation of p53, p21 and bax verified transfection in the current study. However, cyclin D levels decreased following transfection, and the reason for this remains unknown.

Based on the results above, it was concluded that the neddylation pathway may be involved in the development of melanoma. Inhibition of the neddylation pathway affects cell cycle regulators and apoptosis promoters, leading to the depression of melanoma growth. In this study, UBA3 was chosen for neddylation interference. However, UBC12 and APPBP1 were also upregulated in the three melanoma cell lines. In conclusion, proteins which regulate the neddylation pathway may represent potential therapeutic targets in the treatment of melanoma and other tumors associated with neddylation promotion.

## Figures and Tables

**Table I:** Clark levels of seven cases of melanoma.

Group	Gender	Age, years	Region	Clark level
1	Female	50	Big toe	3
2	Female	62	Pelma	4
3	Female	40	Pelma	2
4	Male	71	Pelma	3
5	Male	85	Thigh	4
6	Male	76	Pelma	3
7	Female	66	Heel	4

**Table II:** Percentage of cells in GI, G2+S and apoptosis phases in transfected and non-transfected M14 cells.

Group	G0/G1 (%)	G2+S (%)	Apoptosis (%)
Non-transfected M14	57.4900±4.527^a^	42.4800±4.547^a^	0.3050±0.187^a^
M14/shRNAT-U6.2	59.1325±1.019^a^	40.8725±1.013^a^	0.4375±0.103^a^
M14/shRNA-UBA3	68.3275±1.263	31.6675±1.265	1.2400±0.404

a P<0.05, relative to the M14/shRNA-UBA3 group. Statistical differences were analyzed with the t-test. Data are presented as the mean ± SD (n=4).

**Table III:** Relative density of Bax, P21, P27 and Cyclin D in transfected and non-transfected M14 cells.

Group	bax	p21	p27	cyclin D
Non-transfected M14	0.482	0.572	0.463	0.904
M14/shRNAT-U6.2	0.428	0.490	0.470	0.971
M14/shRNA-UBA3	0.744	0.763	0.653	0.230

## Abbreviations

NEDD8: neural precursor cell-expressed developmentally downregulated 8
